# Associations between endometriosis and adverse pregnancy and perinatal outcomes: a population-based cohort study

**DOI:** 10.1007/s00404-023-07002-y

**Published:** 2023-03-20

**Authors:** Amanuel T. Gebremedhin, Vera R. Mitter, Bereket Duko, Gizachew A. Tessema, Gavin F. Pereira

**Affiliations:** 1 Curtin School of Population Health, Faculty of Health Sciences, Curtin University, Kent Street, GPO Box U1987, Bentley, WA 6102 Australia; 2 PharmacoEpidemiology and Drug Safety Research Group, Department of Pharmacy, and PharmaTox Strategic Research Initiative, Faculty of Mathematics and Natural Sciences, University of Oslo, Oslo, Norway; 3 University Women’s Hospital, Inselspital, Bern University Hospital, University of Bern, Bern, Switzerland; 4 enAble Institute, Curtin University, Kent Street, Bentley, WA 6102 Australia; 5 Centre for Fertility and Health (CeFH), Norwegian Institute of Public Health, Oslo, Norway

**Keywords:** Endometriosis, Preeclampsia, Placenta previa, Preterm birth, Medically assisted reproduction

## Abstract

**Purpose:**

To examine the association between endometriosis and adverse pregnancy and perinatal outcomes (preeclampsia, placenta previa, and preterm birth).

**Methods:**

A population-based retrospective cohort study was conducted among 468,778 eligible women who contributed 912,747 singleton livebirths between 1980 and 2015 in Western Australia (WA). We used probabilistically linked perinatal and hospital separation data from the WA data linkage system’s Midwives Notification System and Hospital Morbidity Data Collection databases. We used a doubly robust estimator by combining the inverse probability weighting with the outcome regression model to estimate adjusted risk ratios (RR) and 95% confidence intervals (CIs).

**Results:**

There were 19,476 singleton livebirths among 8874 women diagnosed with endometriosis. Using a doubly robust estimator, we found pregnancies in women with endometriosis to be associated with an increased risk of preeclampsia with RR of 1.18, 95% CI 1.11–1.26, placenta previa (RR 1.59, 95% CI 1.42–1.79) and preterm birth (RR 1.45, 95% CI 1.37–1.54). The observed association persisted after stratified by the use of Medically Assisted Reproduction, with a slightly elevated risk among pregnancies conceived spontaneously.

**Conclusions:**

In this large population-based cohort, endometriosis is associated with an increased risk of preeclampsia, placenta previa, and preterm birth, independent of the use of Medically Assisted Reproduction. This may help to enhance future obstetric care among this population.

**Supplementary Information:**

The online version contains supplementary material available at 10.1007/s00404-023-07002-y.

## What does this study add to the clinical work

**Table:** 

Women with endometriosis have a greater risk of preeclampsia, placenta previa, and preterm birth, which cannot be explained by the use of medically assisted reproduction. These findings may inform future obstetric care among this population.	

## Introduction

Endometriosis is a chronic inflammatory condition affecting women, where endometrial cells normally lining up the uterine cavity are found outside the uterus. Endometriosis can cause a variety of and sometimes unspecific symptoms with no to severe cyclic pain episodes, dyspareunia, dysmenorrhea, and subfertility [1, 2]. The disease highly affects the quality of life, and productivity, and causes high treatment and societal costs [3]. It often takes 8–12 years from symptom onset to surgical diagnosis [4–6], leading to varying prevalence estimates (5–50% in infertile women, up to 75% in cases with chronic pain) [7, 8]. In Australia, 11% of reproductive-age women are affected with prevalence ranging from 2 to 11% in asymptomatic women [9]. Three-quarters of women with mild to moderate endometriosis can achieve pregnancy spontaneously, despite an increased risk of subfertility [10].

The association between endometriosis and adverse pregnancy outcomes has drawn more attention in recent years with fairly consistent evidence of increased risks for caesarean section, preterm birth, and stillbirth [11, 12]. However, the link with gestational diabetes, preeclampsia, or intrauterine growth restriction remains less clear due to heterogeneity in study designs and methodologies used in previous studies [13–21]. In epidemiology, it remains challenging to study the direct impact of endometriosis on pregnancy outcomes, and underlying mechanisms are not well understood. Much of the existing research on this topic comes from small cohort studies at infertility clinics or single surgical centres [22], which can produce results that are misinterpreted as evidence of no association rather than a lack of evidence for any association. Moreover, data limited to clinical settings are prone to selection bias as these participants may have better access to care, which may be linked to other health behaviours that affect pregnancy outcomes [23]. Classical study designs adopted by studies that do not have access to a wide range of potential risk factors may also be prone to residual confounding.

To address some of these limitations, we used a ‘doubly robust estimator’ to estimate the association between endometriosis and adverse pregnancy outcomes. This approach offers an opportunity to achieve unbiased inference while accounting for selection effects by combining inverse probability weighting and regression adjustment and allows for a causal interpretation of the results [24, 25]. Findings from this approach can be directly interpreted as the risk of adverse pregnancy outcomes given that women had endometriosis as compared to the counterfactual scenario in that they had no endometriosis. This causal interpretation is usually not possible from classical epidemiological approaches. This study aimed to estimate the effect (*average treatment effect)* of endometriosis on adverse pregnancy and perinatal outcomes using a large population-based cohort in Western Australia (WA).

## Methods

### Study design

We conducted a population-based, longitudinal cohort study including all women 15 to 49 years of age with a singleton pregnancy in the period of 1980–2015 in WA.

### Data sources and study population

We obtained maternal, infant and birth information from the Midwives Notification System, a validated database [26] that includes > 99% of births in WA of at least 20 weeks’ gestation or birthweight of 400 g or more if the gestational age was unknown [27]. We sourced hospitalization records from the Hospital Morbidity Data Collection, which includes information on all hospitalizations from public, private and day procedure facilities in the state with International Classification of Diseases (ICD-9/10th revision-Australian Modification) coded diagnoses [28]. Data sources have been described in detail elsewhere [29]. Data were probabilistically linked using best practice protocols through the WA Data Linkage Branch [30].

From a total of 487,297 women (964,015 births) during the study period, we sequentially excluded multiple gestations, stillbirths, and pregnancies with missing information for gestational age, outcomes, maternal age, and socioeconomic status (SES). This resulted in 468,778 eligible women who contributed to 912,747 singleton pregnancies included in the analytic cohort (Fig. S1).

### Exposure assessment

We identified all women with a principal or additional diagnosis of endometriosis from the hospital separation data using the International Classification of Diseases (ICD)-AM (Australian Modification) diagnostic codes consistent with ICD-9: 617.0–617.9; ICD-10: N80.0-N80.9 and Australian Classification of Health Interventions (ACHI) for endometriosis-related procedures (codes are shown in Table S5). Women were categorized as having endometriosis if they had hospital admission or surgical procedure coded as a diagnosis of endometriosis. We included women diagnosed before and after pregnancy in the primary analysis because recent studies documented a diagnosis delay of 8–12 years [4–6]. This approach has been adopted by other recent studies [15, 19].

### Outcomes

The outcomes of interest were ascertained from the Midwives’ Notifications System and hospital separation data in the state, with the diagnostic codes consistent with preeclampsia (ICD-9/ICD-9-CM: 642.4, 642.5, 642.7, ICD-10-AM: O14, O11) and placenta previa, with or without haemorrhage (ICD-9/ICD-9-CM: 641.0–641.1, ICD-10-AM: O44.-). The onset of preeclampsia at the gestational age between 20 and 34 weeks and after 34 weeks of gestation was classified as early or late-onset preeclampsia, respectively. Preterm birth was defined as birth before 37 completed weeks of gestation, categorized into moderate preterm birth (gestational week 32–36) and very preterm birth (prior to 32 gestational weeks). Further, we also categorized preterm birth into spontaneous (due to spontaneous onset of labour) and medically indicated (due to elective caesarean section, or induction of labour). The details of ICD codes used to define variables for analysis are presented in Table S5.

### Covariates

Information on potential confounding factors including the calendar year of birth of the child (categorical variable), maternal age group (15–24, 25–29, 30–34, 35–39, 40–49 years), parity (0, 1, 2,  ≥ 3), smoking during pregnancy (Yes vs No), race/ethnicity (Caucasian versus non-Caucasian), and socioeconomic status (SES) was obtained from the databases. SES was measured using Socio-Economic Indexes for Areas (SEIFA). Specifically, we used the Index of Relative Socio-economic Disadvantage level at the time of birth of the child. These scores were obtained from the Australian Bureau of Statistics [31] and categorized into quintiles.

To assess the potential impact of Medically Assisted Reproduction (MAR) or infertility treatment on perinatal outcomes, we identified pregnancies with MAR procedure (using ACHI) or the following ICD diagnostic codes; ICD-9: 628.0-628.9, V26.1-V26.9, and ICD-10-AM: N97.0-97.9; Z31.1-Z31.9. These codes cover assisted reproductive technology (ART) techniques, intrauterine insemination, and ovulation induction and might include some spontaneously conceived pregnancies in couples with fertility issues [32].

### Statistical analysis

We first estimated the unadjusted relative risks (RRs) with 95% confidence intervals (CI) using generalized linear models (GLM) fitted using a Poisson distribution and a log link function. Next, we estimated the causal effect (*average treatment effect*) of endometriosis on adverse pregnancy outcomes using the potential outcome approach, which allows for the estimation of causal effects in large observational data [33]. This was done by combining the inverse probability of treatment weighting (IPTW) and the outcome regression model in a doubly robust estimation [34]. IPTW weights each person by the inverse of their propensity score. The doubly robust estimation allows us to estimate the unbiased average causal effect when either the outcome regression model (traditional way of obtaining treatment effect) or the propensity score model (treatment selection model) is correctly specified [24, 25]. We estimated the adjusted RRs with 95% CI for each outcome derived using modified Poisson regression with a robust error variance to account for the effects of repeated pregnancies per mother [35]. We fitted the *exposure model* with maternal age, birth year, SES, ethnicity/race, and MAR treatment, and the *outcome model* with maternal age, birth year, SES, ethnicity/race and parity. As preeclampsia and placenta previa may influence the risk of preterm birth, gestational age (< 32, 32–36, > 37 weeks of gestation) was also included in the *exposure* model as a covariate for all outcomes included except for preterm birth. To examine the influence of MAR on the association between endometriosis and adverse pregnancy outcomes, we included a sub-analysis stratified by MAR status (Table 3). To check the covariate balance after propensity score matching using IPTW, we performed diagnostics including standardized differences and % of bias in means of all covariates (Fig. S2, Table S7). The standardized differences describe between-group differences in units of standard deviation. The confounders included in the treatment weighting were decided based on prior knowledge as well as consideration of Directed Acyclic Graphs (DAGs) (Fig. S3).

### Missing data

For the main results, we conducted a complete case analysis as the proportion of missing data was small (< 3%, range 0.6% for gestational age to 1.8% for SES).

### Sensitivity analysis

To check the robustness of our findings, we conducted several sensitivity analyses. Firstly, to ascertain the sensitivity of our result to higher-order parity, we restricted the analysis to primiparous women. Secondly, to limit the possibility of misclassification bias, we conducted an analysis restricted to women (i) with a principal diagnosis of endometriosis, a diagnosis established to be chiefly responsible for occasioning an episode [28]; (ii) with any diagnosis of endometriosis prior to the birth of the child to ensure endometriosis was present during pregnancy; (iii) with endometriosis diagnosis before delivery and up to five years after delivery; and (iv) considering endometriosis diagnosis at more than one-time point during five years look-up period. Thirdly, to explore the potential influence of maternal smoking during pregnancy, which was routinely collected in the Midwifery notification from 1997 onwards, we conducted a separate analysis adjusting for smoking. Next, we compared the effect of endometriosis on preterm birth (very preterm vs moderate). Fifth, we undertake a causal mediation analysis based on the counterfactual framework using a parametric regression approach [36] to estimate the natural direct effect of endometriosis compared with the natural indirect effect through MAR. Finally, to assess the extent of unmeasured confounding, we calculated E-values, which represent the minimum strength of association on the risk ratio scale, that any unmeasured confounder would need to have with both endometriosis and each outcome to fully explain away the observed association, conditional on the measured covariates. [37]

All analyses were performed using Stata version 16.1 (Stata Corporation, College Station, Texas, USA).

## Results

### Cohort characteristics

In total, we included 912,747 eligible singleton births with a gestational age of 20–44 weeks from women (*n* = 468,778) aged 15–49 years in the study period between 1980 and 2015 in WA. In these pregnancies 8874 women (1.9%) had a diagnosis of endometriosis, corresponding to 19,476 pregnancies (2.1%). Women with endometriosis were on average of advanced age at the time of birth (> 35 years), Caucasian, and had a higher proportion of medically assisted reproduction compared to women without endometriosis. Socio-economic status, parity, and ethnicity were similar among exposed and non-exposed groups (Table 1).

**Table 1:** Maternal characteristics according to endometriosis status for women delivering singleton births during 1980–2015 in WA (*n* = 912,747 pregnancies)

Characteristics	Total	Endometriosis	No endometriosis
	*N* = 912,747	(*n* = 19,476)	(*n* = 893,271)
Maternal age, years			
15–24	225,204 (24.7)	4855 (24.9)	220,349 (24.7)
25–29	292,416 (32.0)	6037 (31.0)	286,379 (32.1)
30–34	260,947 (28.6)	5334 (27.4)	255,613 (28.6)
35–39	113,489 (12.4)	2731 (14.0)	110,758 (12.4)
40–49	20,691 (2.3)	519 (2.7)	20,172 (2.3)
SES in quintiles			
< 20th percentile	181,980 (19.9)	3860 (19.8)	178,120 (19.9)
20–39th percentile	182,472 (20.0)	4015 (20.6)	178,457 (20.0)
40–59th percentile	182,685 (20.0)	3765 (19.3)	178,920 (20.0)
60–79th percentile	182,731 (20.0)	3964 (20.4)	178,767 (20.0)
≥ 80th percentile	182,879 (20.0)	3872 (19.9)	179,007 (20.0)
Time period of birth			
1980–1984	102,077 (11.2)	1494 (7.7)	100,583 (11.3)
1985–1989	115,682 (12.7)	2843 (14.6)	112,839 (12.6)
1990–1994	121,186 (13.3)	3791 (19.5)	117,395 (13.1)
1995–1999	121,670 (13.3)	3639 (18.7)	118,031 (13.2)
2000–2004	119,334 (13.1)	2,982 (15.3)	116,352 (13.0)
2005–2009	141,603 (15.5)	2681 (13.8)	138,922 (15.6)
2010–2015	191,195 (20.9)	2046 (10.5)	189,149 (21.2)
Parity			
First birth	368,504 (40.4)	7516 (38.6)	360,988 (40.4)
2nd birth	309,240 (33.9)	6557 (33.7)	302,683 (33.9)
3rd birth	148,061 (16.2)	3375 (17.3)	144,686 (16.2)
≥ 4 birth	86,869 (9.5)	2028 (10.4)	84,841 (9.5)
Missing	73 (0.0)	0 (0.0)	73 (0.0)
MAR			
No	890,327 (97.5)	16,198 (83.2)	874,129 (97.9)
Yes	22,420 (2.5)	3278 (16.8)	19,142 (2.1)
Smoking during pregnancy			
No	427,544 (46.8)	7782 (40.0)	419,762 (47.0)
Yes	80,709 (8.8)	1539 (7.9)	79,170 (8.9)
Missing	404,494 (44.3)	10,155 (52.1)	394,339 (44.1)
Ethnicity (race)			
Caucasian	759,584 (83.2)	17,734 (91.1)	741,850 (83.0)
Non-Caucasian	153,163 (16.8)	1742 (8.9)	151,421 (17.0)

*MAR* medically assisted reproduction, *SES* socio-economic status

The prevalence of pregnancy complications was higher among pregnancies of women with endometriosis compared to women without endometriosis (preeclampsia, 7.5% vs. 6.1%; preterm birth, 10.2% vs. 7.0%; placenta previa, 1.9% vs. 1.0%; Table 2).

**Table 2:** Crude and adjusted Risk ratio (RR) and 95% CI for each adverse pregnancy outcome for women with and without endometriosis among 912,747 singleton births in WA, 1980–2015

Outcomes	No endometriosis *n* = 893,271 (%)	Endometriosis *n* = 19,476 (%)	Unadjusted RR (95% CI)	Adjusted RR (95% CI) using doubly robust estimation
Preeclampsia	54,098 (6.1)	1468 (7.5)	1.24 (1.18, 1.31)	1.18 (1.11, 1.26)
Early	4749 (0.6)	157 (0.8)	1.52 (1.30, 1.78)	1.50 (1.12, 2.02)
Late	32,240 (3.6)	823 (4.3)	1.17 (1.09, 1.25)	1.06 (0.92, 1.21)
Placenta previa	8901 (1.0)	364 (1.9)	1.88 (1.69, 2.08)	1.59 (1.42, 1.79)
Preterm birth	62,706 (7.0)	1989 (10.2)	1.45 (1.39, 1.52)	1.45 (1.37, 1.54)
Spontaneous	36,034 (4.0)	978 (5.0)	1.27 (1.16, 1.38)	1.40 (1.27, 1.56)
Indicated	26,671 (3.0)	1011 (5.2)	1.62 (1.48, 1.78)	1.74 (1.58, 1.93)

Doubly robust estimation: the *exposure* model included maternal age, birth year, SES, ethnicity/race, and MAR treatment, and the outcome model included maternal age, birth year, SES, parity, and ethnicity/race. The outcome model for preeclampsia and placenta previa also included gestational age

*SES* socio-economic status, *MAR* medically assisted reproduction, *RR* risk ratio, *CI* confidence interval, *n* total number of pregnancies from women with endometriosis

Using doubly robust estimation, pregnancies in women with a diagnosis of endometriosis were associated with a higher risk of preeclampsia (RR 1.18, 95% CI 1.11–1.26), placenta previa (RR 1.59, 95% CI 1.42–1.79), and preterm birth (RR 1.45, 95% CI 1.37–1.54). This risk associated with endometriosis was higher for medically indicated preterm birth (RR 1.74, 95% CI 1.58–1.93) compared to spontaneous preterm birth (RR 1.40, 95% CI 1.27–1.56) (Table 2). Furthermore, endometriosis was associated with both moderate and very preterm birth with a stronger association observed for very preterm birth (Table S3). In a stratified analysis based on MAR status, the higher risk of placenta previa and preterm birth persisted regardless of conception mode with the strongest effect estimates for the non-MAR group (RR 1.77, 95% CI 1.50–2.08 for placenta previa and RR 1.67, 95% CI 1.55–1.80 for preterm birth) and slightly attenuated effect estimates among the MAR group. However, for preeclampsia, the observed association disappeared when stratified by MAR status (Table 3).

**Table 3:** Crude and adjusted Risk ratio (RR) and 95% CI for each adverse pregnancy outcome for women with and without endometriosis stratified by MAR status among 912,747 singleton births in WA, 1980–2015

Outcome		MAR (*n* = 22,420)				Non-MAR (*n* = 890,327)				*P*-value
		Number	PE (%)	Crude RR (95% CI)	Adjusted RR (95% CI)	Number	PE (%)	Crude RR (95% CI)	Adjusted RR (95% CI)	
PE	Endometriosis									< 0.001
	Yes	3278	214 (6.5)	0.99 (0.86, 1.14)	0.86 (0.69, 1.07)	16,198	1254 (7.7)	1.28 (1.21, 1.35)	1.22 (1.10, 1.35)	
	No	19,142	1256 (6.6)			874,129	52, 842 (6.0)			
PP		Number	PP (%)	Crude RR (95% CI)	Adjusted RR (95% CI)	Number	PP (%)	Crude RR (95% CI)	Adjusted RR (95% CI)	< 0.001
	Endometriosis									
	Yes	3278	82 (2.5)	1.20 (0.95, 1.52)	1.11 (0.83, 1.46)	16,198	282 (1.7)	1.79 (1.59, 2.01)	1.77 (1.50, 2.08)	
	No	19,142	400 (2.1)			874,129	8501 (1.0)			
PTB		Number	PTB (%)	Crude RR (95% CI)	Adjusted RR (95% CI)	Number	PTB (%)	Crude RR (95% CI)	Adjusted RR (95% CI)	< 0.001
	Endometriosis									
	Yes	3278	416 (12.7)	1.21 (1.09, 1.33)	1.26 (1.09, 1.45)	16,198	1573 (9.7)	1.40 (1.33, 1.47)	1.67 (1.55, 1.80)	
	No	19,142	2011 (10.5)			874,129	60,695 (6.9)			

All adjusted RRs presented in this table are estimated using a doubly robust estimation: the *exposure* model included maternal age, birth year, SES, and ethnicity/race, and the *outcome* model included maternal age, birth year, SES, parity, and ethnicity/race. The *outcome* model for preeclampsia and placenta previa also included gestational age

*PE* preeclampsia, *PP* placenta previa, *PTB* Preterm birth, *SES* socio-economic status, *MAR* medically assisted reproduction, *RR* risk ratio, *CI* confidence interval, *n* total number of pregnancies from women with endometriosis

^a^*P*-value for interaction test

### Results of the sensitivity analyses

The results restricted to nulliparous women were consistent with the main finding with a slight attenuation (Table S2; Model 2). Findings from the analyses restricted to a subset of the study population with different exposure definitions were very similar to those reported in the main analyses (Table S2; Model 3–6). Analyses restricted to pregnancies from women with endometriosis diagnosed before delivery also resulted in slightly higher risk estimates for all adverse pregnancy outcomes evaluated (Table S2; Model 4). Additionally, the pattern of the association between endometriosis and adverse pregnancy outcomes was similar when further adjusted to smoking status (Table S2; Model 7). Our mediation analyses suggest that the percentage of endometriosis effect on preterm birth and placenta previa that was mediated through MAR was 8% and 3% respectively. (Table S4). The E-values for the observed RRs varied from 1.64 to 2.87 for these three adverse pregnancy outcomes (Table S6).

## Discussion

### Principal findings

To our knowledge, this is the first population-based retrospective cohort study to examine the association between endometriosis with adverse pregnancy outcomes using the potential outcome framework. Using a large (~ 1 million births) cohort in WA, we observed a higher risk of preeclampsia, placenta previa, and preterm birth among pregnancies in women with endometriosis as compared to women without endometriosis. The associations persisted after stratification for conception mode (MAR or natural conception, non-MAR), with an elevated risk among the non-MAR group meaning the risks observed were attenuated among pregnancies conceived by MAR. The risk for adverse pregnancy outcomes was higher (approximately 24%, 56%, and 85% of increased risk of preeclampsia, preterm birth, and placenta previa, respectively) when we restricted our sample to women with endometriosis as the principal diagnosis code, suggesting probably more severe disease. These observed associations were not mediated through MAR.

### Strengths and limitations

Our cohort was based on longitudinally linked, highly reliable sources of population-based perinatal information ascertained from hospital separations and midwives’ notifications. We also included sensitivity analysis to check the robustness of our result. Our cohort is less prone to exposure misclassification bias since the hospital morbidity data collection (source data for our exposure) contains records for all hospital separations of admitted patients from all public and private hospitals in WA. Furthermore, our study restricted the analysis to singleton pregnancies, which improved generalizability to other similar cohorts.

We only had information on the diagnosis of endometriosis for women who have been hospitalized during the study period. Such data may likely represent more severe stages of endometriosis. The clinical routines and obstetric care have changed through the years and the diagnosis and awareness regarding endometriosis have evolved. However, to minimize this bias our model included the birth year of the child as a covariate. In our main analysis, we included all women with any diagnosis of endometriosis (principal and additional diagnosis). This could have introduced non-differential misclassification bias (i.e., independent of the outcome) and, therefore, will potentially bias the results towards the null. To limit this possible misclassification, we included a sensitivity analysis restricted to an exposure defined as a principal diagnosis of endometriosis, which indicated higher risk estimates as compared to the main result. We opted to include women with a diagnosis of endometriosis before and after pregnancy to account for the diagnostic delay [4, 5]. This could induce similar misclassification bias and attenuation of the association. Indeed, our sensitivity analysis restricted to a diagnosis of endometriosis before delivery consistently suggested higher effect estimates as compared to the main analysis. Though the validity of the diagnosis of endometriosis in the hospital separation database remains unknown, previous analysis of the same database suggested that endometriosis is reliably recorded in the hospital separation data [38]. Moreover, while the use of ICD and procedure codes ensures that those classified as having endometriosis are likely true cases, there is the possibility that the comparison group may have undiagnosed endometriosis. Our cohort had a relatively small number of events for stillbirths to be considered as an outcome. We, therefore, excluded pregnancies resulting in stillbirths from our analysis. This may have introduced a livebirth bias in the association between endometriosis and adverse pregnancy outcomes. However, a previous simulation study indicated that the magnitude of this bias is small [39]. Despite the potential susceptibility of doubly robust estimation to the limitations of misspecification bias, our study employs directed acyclic graphs (DAGs) in the selection of variables for the exposure and outcome models which helps to address the limitation and strengthens the robustness of our results.

### Interpretation

Our study observed a modest association between endometriosis and preeclampsia, which is consistent with previous studies [12, 15, 20, 21]. However, other studies have reported a lower risk of preeclampsia [13, 17] with some suggesting no association [14]. These controversial results may be related to sample size, heterogeneity in exposure or outcome definition, selection bias, not taking MAR into account, diagnostic methods, and disease heterogeneity.

The attenuated risk in women who received MAR treatment is supported by other studies that did not find an association or a reduced association between endometriosis and preeclampsia or hypertension in pregnancy in women with an endometriosis and MAR procedure. [40, 41] Our study also found an increased risk of placenta previa in pregnancies among women with endometriosis, which is consistent with other research [20]. It has been suggested that the association may be confounded by the increased use of MAR in women with endometriosis. In our study, the association persisted even after stratification by MAR status, with a stronger association observed in non-MAR women which is consistent with other studies [40]. In our study, for women using MAR, the precision of the effect estimates was reduced likely because of the small sample size. For preterm birth, we observed higher risk estimates for very preterm deliveries compared to moderate preterm deliveries, and an association between endometriosis and both spontaneous and medically indicated preterm birth, with a stronger association for medically indicated preterm birth. This could imply that pregnancies from women with endometriosis are more likely to be induced or delivered through a caesarean section before gestational week 37. This finding is consistent with previous research and the association seems independent of MAR. [20, 41, 42] A smaller protective effect was also observed in a Canadian study [12].

Endometriosis may be associated with adverse pregnancy outcomes through various mechanisms, including effects on the uterine environment, progesterone signalling, and the remodelling of the spiral artery [18, 43, 44]. These factors may play a role in the association with preeclampsia, preterm birth and intrauterine growth restriction [45–47]. Endometriotic lesions in the uterus may also reduce uterine contractility and cause abnormal implantation, leading to placenta previa [48]. In our sub-analysis of the timing of preeclampsia, we found an elevated risk for early onset compared to late-onset preeclampsia, which can be accounted for inadequate and incomplete trophoblast invasion of maternal spiral arteries [43]. MAR treatment itself has shown to be a risk factor for adverse pregnancy outcomes, with mixed results for women with endometriosis [47]. In MAR treatment, the effects caused by endometriosis such as inflammatory processes and regulatory disbalances are suppressed offering a better pregnancy environment and could explain the attenuation in the risks seen in our study in the MAR pregnancies group [49]. Women conceiving following MAR might also have support from better obstetric care and closer screening for adverse outcomes.

In this study, a stronger association between endometriosis and adverse pregnancy outcomes was observed, but residual and/or unmeasured confounding could not be completely ruled out. Nevertheless, the E-values for the observed RRs (ranging from 1.64 to 2.87) indicated that substantial confounding would need to explain away these associations (Table S6). Systematic reviews that examined the risk factors for endometriosis, for example, reported RRs ranging from 1.63 for smoking to 1.87 for overweight—lower than that of the *E*-values. [50, 51] In general, findings from our sensitivity analyses were remarkably similar to those reported for the main analysis and collectively support the hypothesis that endometriosis is associated with adverse pregnancy outcomes independent of MAR. Therefore, knowledge of a patient's endometriosis history may inform targeted prenatal care and reduce unfavourable pregnancy outcomes. Future studies would benefit from elucidating the potential mechanism that might explain how endometriosis affects implantation, placentation, and fetal growth and identifying potential interventions to decrease the risk of adverse perinatal outcomes.

## Conclusion

In conclusion, regardless of the use of medically assisted reproduction, endometriosis is associated with an increased risk of preeclampsia, placenta previa, and preterm birth. These findings offer new insight into the causal association between endometriosis on adverse pregnancy outcomes, taking MAR into account. This may help to enhance future obstetric care among this population.

### Supplementary Information

Below is the link to the electronic supplementary material.

Supplementary file1 (DOCX 7038 KB)

## Data Availability

No additional data are available.
