# Supplementary material for: Associations between endometriosis and adverse pregnancy and perinatal outcomes: a population-based cohort study
Source: Arch Gynecol Obstet. 2023 Mar 20;309(4):1323–31. doi: 10.1007/s00404-023-07002-y (PMC10894157; doi:10.1007/s00404-023-07002-y)
Supplement: Supplementary file 1 — Supplementary file1 (DOCX 7038 KB) [file 404_2023_7002_MOESM1_ESM.docx]

**Supplementary appendix**

**Authors:** Amanuel T GEBREMEDHIN,^1, *^ Vera R MITTER,^2,3^ Bereket DUKO,^1^ Gizachew A TESSEMA,^1^ Gavin F PEREIRA^1,4,5^

**Author Affiliations:**

^1^ Curtin School of Population Health, Faculty of Health Sciences, Curtin University, Kent Street, Bentley WA 6102, Australia

^2^ PharmacoEpidemiology and Drug Safety Research Group, Department of Pharmacy, and PharmaTox Strategic Research Initiative, Faculty of Mathematics and Natural Sciences, University of Oslo, Oslo, Norway.

^3^University Women's Hospital, Inselspital, Bern University Hospital, University of Bern, Bern, Switzerland.

^4^enAble Institute, Curtin University, Kent Street, Bentley, Western Australia 6102, Australia

^5^Centre for Fertility and Health (CeFH), Norwegian Institute of Public Health, Oslo, Norway

**^*^ Corresponding Author:**

Dr Amanuel Tesfay Gebremedhin

Curtin School of Population Health, Curtin University

GPO Box U1987, Perth Western Australia 6845

Email: a.gebremedhin@curtin.edu.au

ORCID: 0000-0003-2459-1805


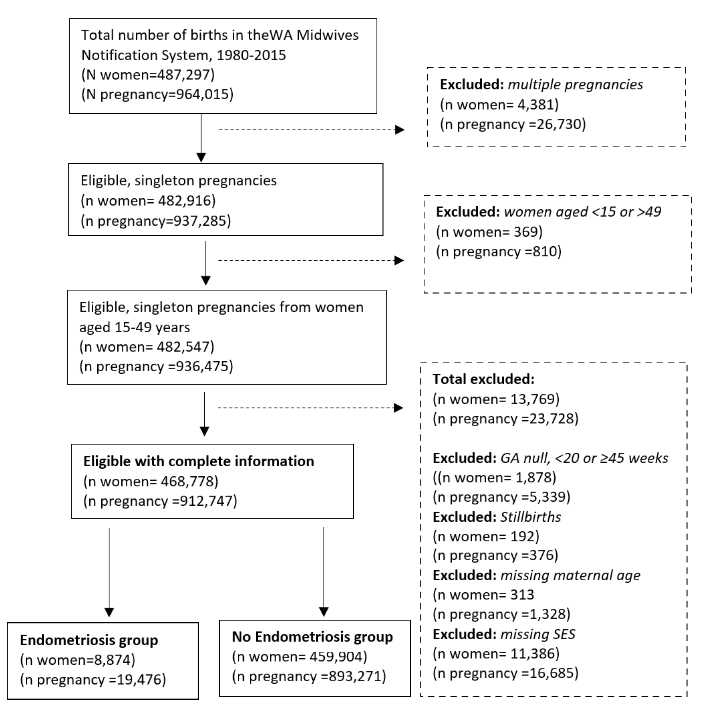


**Fig. S1 Inclusions and exclusions for the study cohort**


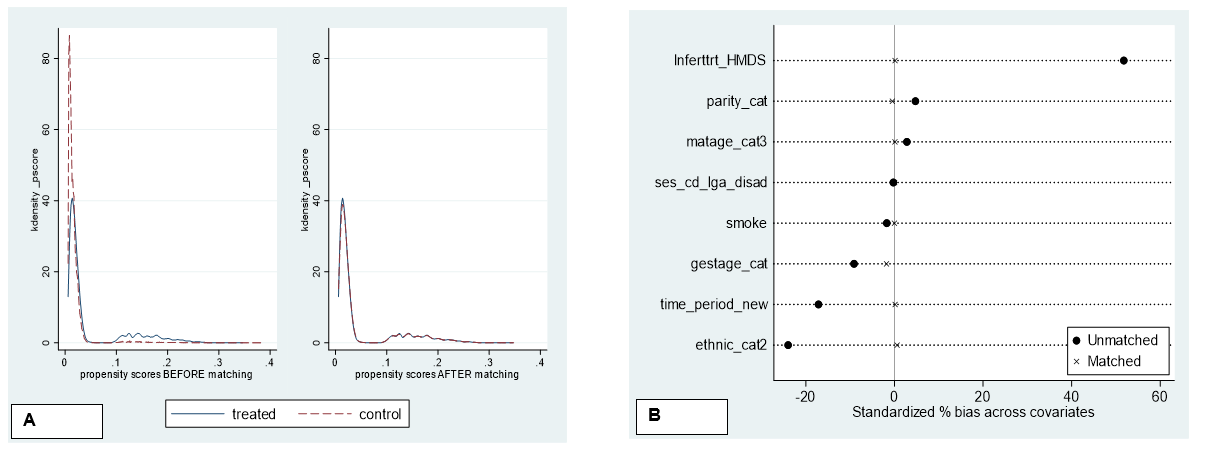


**Fig. S2 Covariate balance before and after propensity-score matching (unmatched vs matched) using (a)density graph and (b) standardized % of bias across covariates.** Standardized differences are calculated as the difference in means between treatment and control samples divided by the standard deviation of the treatment sample for each covariate. The standardized difference will approach zero when the distribution for a particular covariate is more similar between treatment (endometriosis) and control (no endometriosis) samples.


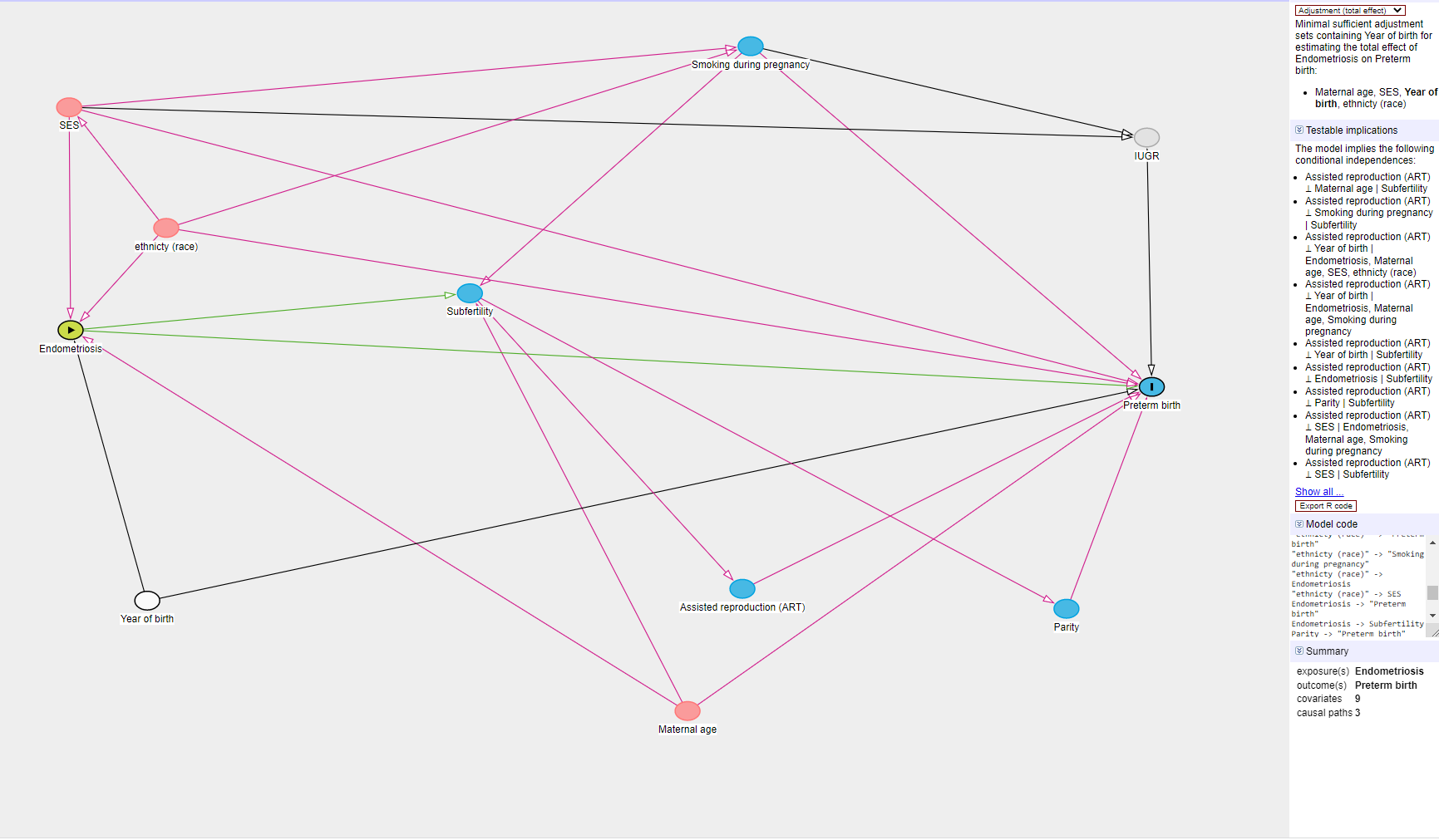


**Fig. S3** **Directed acyclic graph (DAG) representing the causal association between endometriosis and preterm birth**

Outcome: Preterm birth; exposure: endometriosis. We assume that maternal age, birth year, SES, parity, Smoking, and ethnicity (race) are directly associated with the outcome. While, maternal age, birth year, SES, MAR, and ethnicity (race) are associated with the exposure.

**Table S1. Maternal characteristics by the outcome of interest for women delivering singleton infants during 1980-2015 in WA (n=912,747 pregnancies)**

|  |  | |  | | **Outcomes** | | | | | | | | | |
| --- | --- | --- | --- | --- | --- | --- | --- | --- | --- | --- | --- | --- | --- | --- |
| **Characteristics** |  | |  | | **PE** | |  | | **PTB** | | | **Placenta previa** | | |
|  |  | **Total** | | **No** | | **Yes** | | **No** | | **Yes** | **No** | | **Yes** |  |
|  |  | N=912,747 | | N=857,181 | | N=55,566 | | N=848,052 | | N=64,695 | N=903,482 | | N=9,265 |  |
| Endometriosis |  | 19,476 (2.1) | | 18,008 (2.1) | | 1,468 (2.6) | | 17,487 (2.1) | | 1,989 (3.1) | 19,112 (2.1) | | 364 (3.9) |  |
| Maternal age, y | 15-24 | 225,204 (24.7) | | 208,304 (24.3) | | 16,900 (30.4) | | 207,378 (24.5) | | 17,826 (27.6) | 224,023 (24.8) | | 1,181 (12.7) |  |
|  | 25-29 | 292,416 (32.0) | | 274,518 (32.0) | | 17,898 (32.2) | | 273,534 (32.3) | | 18,882 (29.2) | 289,891 (32.1) | | 2,525 (27.3) |  |
|  | 30-34 | 260,947 (28.6) | | 247,375 (28.9) | | 13,572 (24.4) | | 243,685 (28.7) | | 17,262 (26.7) | 257,749 (28.5) | | 3,198 (34.5) |  |
|  | 35-39 | 113,489 (12.4) | | 107,603 (12.6) | | 5,886 (10.6) | | 104,750 (12.4) | | 8,739 (13.5) | 111,613 (12.4) | | 1,876 (20.2) |  |
|  | 40-49 | 20,691 (2.3) | | 19,381 (2.3) | | 1,310 (2.4) | | 18,705 (2.2) | | 1,986 (3.1) | 20,206 (2.2) | | 485 (5.2) |  |
| SES in quintiles | <20th percentile | 181,980 (19.9) | | 169,716 (19.8) | | 12,264 (22.1) | | 166,582 (19.6) | | 15,398 (23.8) | 180,321 (20.0) | | 1,659 (17.9) |  |
|  | 20-39th percentile | 182,472 (20.0) | | 170,348 (19.9) | | 12,124 (21.8) | | 169,353 (20.0) | | 13,119 (20.3) | 180,724 (20.0) | | 1,748 (18.9) |  |
|  | 40-59th percentile | 182,685 (20.0) | | 171,247 (20.0) | | 11,438 (20.6) | | 170,200 (20.1) | | 12,485 (19.3) | 180,910 (20.0) | | 1,775 (19.2) |  |
|  | 60-79th percentile | 182,731 (20.0) | | 172,193 (20.1) | | 10,538 (19.0) | | 170,507 (20.1) | | 12,224 (18.9) | 180,798 (20.0) | | 1,933 (20.9) |  |
|  | >=80th percentile | 182,879 (20.0) | | 173,677 (20.3) | | 9,202 (16.6) | | 171,410 (20.2) | | 11,469 (17.7) | 180,729 (20.0) | | 2,150 (23.2) |  |
| Time period of births | 1980-1984 | 102,077 (11.2) | | 93,776 (10.9) | | 8,301 (14.9) | | 95,308 (11.2) | | 6,769 (10.5) | 101,279 (11.2) | | 798 (8.6) |  |
|  | 1985-1989 | 115,682 (12.7) | | 106,369 (12.4) | | 9,313 (16.8) | | 107,915 (12.7) | | 7,767 (12.0) | 114,719 (12.7) | | 963 (10.4) |  |
|  | 1990-1994 | 121,186 (13.3) | | 112,056 (13.1) | | 9,130 (16.4) | | 112,924 (13.3) | | 8,262 (12.8) | 120,090 (13.3) | | 1,096 (11.8) |  |
|  | 1995-1999 | 121,670 (13.3) | | 111,916 (13.1) | | 9,754 (17.6) | | 113,113 (13.3) | | 8,557 (13.2) | 120,415 (13.3) | | 1,255 (13.5) |  |
|  | 2000-2004 | 119,334 (13.1) | | 112,340 (13.1) | | 6,994 (12.6) | | 110,681 (13.1) | | 8,653 (13.4) | 117,914 (13.1) | | 1,420 (15.3) |  |
|  | 2005-2009 | 141,603 (15.5) | | 135,926 (15.9) | | 5,677 (10.2) | | 131,018 (15.4) | | 10,585 (16.4) | 139,918 (15.5) | | 1,685 (18.2) |  |
|  | 2010-2015 | 191,195 (20.9) | | 184,798 (21.6) | | 6,397 (11.5) | | 177,093 (20.9) | | 14,102 (21.8) | 189,147 (20.9) | | 2,048 (22.1) |  |
| Parity | first birth | 368,504 (40.4) | | 335,184 (39.1) | | 33,320 (60.0) | | 340,383 (40.1) | | 28,121 (43.5) | 365,310 (40.4) | | 3,194 (34.5) |  |
|  | 2^nd^ birth | 309,240 (33.9) | | 296,667 (34.6) | | 12,573 (22.6) | | 290,499 (34.3) | | 18,741 (29.0) | 305,887 (33.9) | | 3,353 (36.2) |  |
|  | 3^rd^ birth | 148,061 (16.2) | | 142,184 (16.6) | | 5,877 (10.6) | | 138,329 (16.3) | | 9,732 (15.0) | 146,491 (16.2) | | 1,570 (16.9) |  |
|  | >=4 birth | 86,869 (9.5) | | 83,086 (9.7) | | 3,783 (6.8) | | 78,773 (9.3) | | 8,096 (12.5) | 85,721 (9.5) | | 1,148 (12.4) |  |
|  | Missing | 73 (0.0) | | 60 (0.0) | | 13 (0.0) | | 68 (0.0) | | 5 (0.0) | 73 (0.0) | | 0 (0.0) |  |
| MAR | No | 890,327 (97.5) | | 836,231 (97.6) | | 54,096 (97.4) | | 828,059 (97.6) | | 62,268 (96.2) | 881,544 (97.6) | | 8,783 (94.8) |  |
|  | Yes | 22,420 (2.5) | | 20,950 (2.4) | | 1,470 (2.6) | | 19,993 (2.4) | | 2,427 (3.8) | 21,938 (2.4) | | 482 (5.2) |  |
| Smoking during pregnancy | No | 427,544 (46.8) | | 407,502 (47.5) | | 20,042 (36.1) | | 398,541 (47.0) | | 29,003 (44.8) | 422,696 (46.8) | | 4,848 (52.3) |  |
|  | Yes | 80,709 (8.8) | | 77,470 (9.0) | | 3,239 (5.8) | | 72,495 (8.5) | | 8,214 (12.7) | 79,752 (8.8) | | 957 (10.3) |  |
|  | Missing | 404,494 (44.3) | | 372,209 (43.4) | | 32,285 (58.1) | | 377,016 (44.5) | | 27,478 (42.5) | 401,034 (44.4) | | 3,460 (37.3) |  |
| Ethnicity (race) | Caucasian | 759,584 (83.2%) | | 711,676 (83.0%) | | 47,908 (86.2%) | | 709,573 (83.7%) | | 50,011 (77.3%) | 751,884 (83.2%) | | 7,700 (83.1%) |  |
|  | Non-Caucasian | 153,163 (16.8%) | | 145,505 (17.0%) | | 7,658 (13.8%) | | 138,479 (16.3%) | | 14,684 (22.7%) | 151,598 (16.8%) | | 1,565 (16.9%) |  |

PE: preeclampsia; PP: placenta previa; PTB: Preterm birth; MAR: Medically Assisted Reproduction; SES: Socio-economic status

**Table S2. Crude and adjusted Risk ratio (RR) and 95 % CI for each adverse pregnancy outcome by endometriosis exposure according to different scenarios (sensitivity analyses) among singleton births in WA, 1980-2015**

| **VARIABLES** | **Unadjusted** | **Model 1** | **Model 2** | **Model 3** | **Model 4** | **Model 5** | **Model 6** | **Model 7** |  |  |  |  |  |
| --- | --- | --- | --- | --- | --- | --- | --- | --- | --- | --- | --- | --- | --- |
|  | **Crude RR (95 % CI)** | **Adjusted RR (95 % CI) ^a^** | **Adjusted RR (95 % CI) ^b^** | **Adjusted RR (95 % CI) ^c^** | **Adjusted RR (95 % CI) ^d^** | **Adjusted RR**  **(95 % CI) ^e^** | **Adjusted RR**  **(95 % CI) ^f^** | **Adjusted RR**  **(95 % CI) ^g^** |  |  |  |  |  |
| **Preeclampsia** | | | | | | |  |  |  |  |  |  |  |
| Endometriosis | 1.24 | 1.18 | 1.14 | 1.24 | 1.37 | 1.13 | 1.47 | 1.14 |  |  |  |  |  |
|  | (1.18 - 1.31) | (1.11 - 1.26) | (1.06 - 1.23) | (1.14 - 1.35) | (1.09 - 1.72) | (1.00 – 1.27) | (1.15- 1.88) | (1.03- 1.26) |  |  |  |  |  |
| **Preterm birth** | | | | | | |  |  |  |  |  |  |  |
| Endometriosis | 1.45 | 1.45 | 1.32 | 1.56 | 1.61 | 1.62 | 1.95 | 1.57 |  |  |  |  |  |
|  | (1.37 - 1.54) | (1.37 - 1.54) | (1.22 - 1.42) | (1.44 - 1.68) | (1.39- 1.86) | (1.48 – 1.76) | (1.61 – 2.36) | (1.45 - 1.69) |  |  |  |  |  |
| **Placenta previa** | | | | | | |  |  |  |  |  |  |  |
| Endometriosis | 1.88 | 1.60 | 1.69 | 1.85 | 1.59 | 1.63 | 2.15 | 1.67 |  |  |  |  |  |
|  | (1.69 - 2.08) | (1.42 - 1.79) | (1.39 - 2.05) | (1.60 - 2.15) | (1.22 - 2.06) | (1.38 – 1.92) | (1.54 -3.00) | (1.43 - 1.94) |  |  |  |  |  |
| **N** | **912,747** | **912,747** | **368,504** | **912,747** | **508,253** | **508,253** | **508,253** | **508,253** |  |  |  |  |  |
| n (endometriosis) | 19,476 | 19,476 | 7,516 | 10,075 | 3,113 | 11,995 | 2,182 | 9,321 |  |  |  |  |  |

All adjusted RRs (Model 1 to Model 5) presented in this table are estimated using a doubly robust estimation with the *exposure* model including maternal age, birth year (continuous), SES, ethnicity/race, and MAR treatment, and the *outcome* model including maternal age, birth year (continuous), SES, parity, and ethnicity/race. The outcome model for preeclampsia and placenta previa also included gestational age. ^a^ includes all pregnancies without any restriction; ^b^ restricted to nulliparous women; ^c^ analysis from all pregnancies with a principal diagnosis of endometriosis; ^d^ analysis restricted to all pregnancies with any diagnosis of endometriosis before delivery; ^e^ analysis restricted to all pregnancies with an endometriosis diagnosis before delivery and up to five years after delivery; ^f^  analysis restricted to all pregnancies with an endometriosis diagnosis in the five years look up period with coding for endometriosis considered at more than one time point during the follow-up; ^g^ Model 1 plus smoking in the *outcome* model. *SES: Socio-economic status; MAR: Medically Assisted Reproduction; RR: Risk Ratio; CI: Confidence interval; n: Total number of pregnancies from women with endometriosis*

**Table S3. Crude and adjusted Risk ratio (RR) and 95 % CI for moderate versus very preterm birth for women with and without endometriosis among 912,747 singleton births in WA, 1980-2015**

| Outcomes | | No endometriosis (n=893,271) | Endometriosis (n=19,476) | Unadjusted RR (95 % CI) | Adjusted RR (95 % CI) using doubly robust estimation | | |
| --- | --- | --- | --- | --- | --- | --- | --- |
| Preterm | | 62,706 (7.0) | 1,989 (10.2) | 1.45 (1.39, 1.52) | | 1.45 (1.37, 1.54) |  |
|  | Moderate | 26,314 (2.9) | 788 (4.0) | 1.37 (1.28, 1.47) | | 1.36 (1.26, 1.48) |  |
|  | Very | 11,087 (1.2) | 412 (2.1) | 1.70 (1.55, 1.88) | | 1.55 (1.39, 1.73) |  |

Doubly robust estimation: the *exposure* model included maternal age, birth year (continuous), SES, ethnicity/race, and MAR treatment, and the *outcome* model included maternal age, birth year (continuous), SES, parity, and ethnicity/race. *SES: Socio economic status; MAR: Medically Assisted Reproduction; RR: Risk Ratio; CI: Confidence interval*

**Table S4. Causal Mediation Analysis of the association between endometriosis and pregnancy outcomes [Relative risk (RR) (95% CI]**

| Effect | PE | PP | PTB |
| --- | --- | --- | --- |
|  | RR (95% CI) | | |
| Natural direct effect (NDE) | 1.19 (1.09 -1.31) | 1.73 (1.48 -2.04) | 1.56 (1.46 -1.68) |
| Natural Indirect Effect (NIE) | 0.96 (0.93 - 0.98) | 1.01 (0.96-1.07) | 1.03 (1.01-1.06) |
| Marginal total effect | 1.15 (1.05 -1.25) | 1.76 (1.53 -2.03) | 1.62 (1.52 -1.72) |
| Proportion mediated by MAR =(NDE*(NIE-1)) / (NDE*NIE -1) | a | 0.03 (3%) | 0.08 (8%) |

The causal mediation analyses estimate two models: a model for MAR (mediator) conditional on endometriosis(exposure) and covariates, and a model for each outcome conditional on exposure, the mediator, and covariates. Covariates were maternal age, birth year (continuous), SES, parity, smoking, and ethnicity/race. *SES: Socio economic status; MAR: Medically Assisted Reproduction; RR: Risk Ratio; CI: Confidence interval; ^a^* small protective effect of mediator observed.

**Table S5. ICD-9/10 codes used to define variables for analysis**

| **ICD-9 code** | **ICD-10** | **code description** | **Type** |
| --- | --- | --- | --- |
| **Preeclampsia** | | | |
| 642.7 | O11 | Preeclampsia with pre-existing hypertension | Any diagnosis; principal diagnosis |
| 642.4-642.5 | O14 | Gestational hypertension with significant proteinuria | Any diagnosis; principal diagnosis |
| **Placenta previa** | | | |
| 641.0- 641.1 | O44 | Placenta previa with or without hemorrhage | Any diagnosis; principal diagnosis |
| **Endometriosis** | | | |
| 617.0 | N80.0 | Endometriosis of the uterus (includes adenomyosis) | Any diagnosis; principal diagnosis |
| 617.1 | N80.1 | Endometriosis of ovary | Any diagnosis; principal diagnosis |
| 617.2 | N80.2 | Endometriosis of fallopian tube | Any diagnosis; principal diagnosis |
| 617.3 | N80.3 | Endometriosis of pelvic peritoneum | Any diagnosis; principal diagnosis |
| 617.4 | N80.4 | Endometriosis of rectovaginal septum and vagina | Any diagnosis; principal diagnosis |
| 617.5 | N80.5 | Endometriosis of intestine | Any diagnosis; principal diagnosis |
| 617.6 | N80.6 | Endometriosis in cutaneous scar | Any diagnosis; principal diagnosis |
| 617.8 | N80.8 | Other endometriosis | Any diagnosis; principal diagnosis |
| 617.9 | N80.9 | Endometriosis, unspecified | Any diagnosis; principal diagnosis |
| **Treatment of infertility (Assisted Reproductive Technology)** | | | |
| 628.0 | N97.0 | Female infertility associated with anovulation | Any diagnosis; principal diagnosis |
| 628.2 | N97.1 | Female infertility of tubal origin | Any diagnosis; principal diagnosis |
| 628.3 | N97.2 | Female infertility of uterine origin | Any diagnosis; principal diagnosis |
| 628.4 | N97.3 | Female infertility of cervical origin | Any diagnosis; principal diagnosis |
| 628.4 | N97.4 | Female infertility associated with male factors | Any diagnosis; principal diagnosis |
| 628.4; 628.8 | N97.8 | Female infertility of other origins | Any diagnosis; principal diagnosis |
| 628.9^¥^ | N97.9^¥^ | Female infertility, unspecified | Any diagnosis; principal diagnosis |
| V26.1 | Z31.1 | Artificial insemination | Any diagnosis; principal diagnosis |
| - | Z31.2^¥^ | In vitro fertilization | Any diagnosis; principal diagnosis |
| - | Z31.3 | Other assisted fertilization methods (including ICSI) | Any diagnosis; principal diagnosis |
| V26.2 | Z31.4 | Procreative investigation and testing | Any diagnosis; principal diagnosis |
| V26.3 | Z31.5 | Genetic counseling | Any diagnosis; principal diagnosis |
| V26.4 | Z31.6 | General counseling and advice on procreation | Any diagnosis; principal diagnosis |
| V26.8 | Z31.8 | Other procreative management | Any diagnosis; principal diagnosis |
| V26.9 | Z31.9 | Procreative management, unspecified | Any diagnosis; principal diagnosis |
| V26.5 | - | Sterilization status | Any diagnosis; principal diagnosis |
| **ACHI codes with description for endometriosis-related procedures** | | | |
| 35630-00: Diagnostic hysteroscopy | | | |
| 35640-00: Dilation and curettage of uterus [D&C] | | | |
| 35637-10: Laparoscopic excision of lesion of pelvic cavity | | | |
| 30393-00: Laparoscopic division of abdominal adhesions | | | |
| 35637-02: Laparoscopic diathermy of lesion of pelvic cavity | | | |
| 35703-00: Test for tubal patency | | | |
| 35503-00: Insertion of intrauterine devic [IUD] | | | |
| 36812-00: Cytoscopy | | | |
| 95550-03: Allied health intervention, physiotherapy | | | |
| 35638-04: Laparoscopic ovarian cystectomy, unilateral | | | |
| **Procedure (Australian Classification of Health Intervention ;ACHI code with description) for MAR** | | | |
| 13212-00: Transvaginal oocyte retrieval | | | |
| 13212-01: Transabdominal oocyte retrieval | | | |
| 13215-00: Gamete intrafallopian transfer [GIFT] | | | |
| 13215-01: Embryo transfer to uterus | | | |
| 13215-02: Embryo transfer to fallopian tube | | | |
| 13218-00: Fresh, frozen, thawed embryo transfer to uterus | | | |
| 13218-01: Fresh, frozen, thawed embryo transfer to Fallopian tube | | | |
| 13251-00: Intracytoplasmic sperm injection (ICSI) | | | |

^¥^ Among the cohort with MAR diagnosis, the following were the major ICD codes: N97.9, Z31.2, 628.9 with 47%, 38% and 21% respectively: all others in the table contributed <5%.The proportion is not mutually exclusive.

**Table S6. E-values to quantify the minimum strength of association that unmeasured confounders would need to have with both endometriosis and each outcome, conditional on the measured covariates, to fully explain the observed association between endometriosis and pregnancy outcomes.**

| Outcomes | | No endometriosis (n=893,271) | Endometriosis (n=19,476) | Unadjusted RR (95 % CI) | Adjusted RR  (95 % CI) | E-value for RR (E- value for CI) |
| --- | --- | --- | --- | --- | --- | --- |
| Preeclampsia | | 54,098 (6.1) | 1,468 (7.5) | 1.24 (1.18, 1.31) | 1.18 (1.11, 1.26) | 1.64 (1.46) |
| Placenta previa | | 8,901 (1.0) | 364 (1.9) | 1.88 (1.69, 2.08) | 1.60 (1.42, 1.79) | 2.58 (2.19) |
| Preterm | | 62,706 (7.0) | 1,989 (10.2) | 1.45 (1.39, 1.52) | 1.45 (1.37, 1.54) | 2.26 (2.08) |
|  | Spontaneous | 36,034 (4.0) | 978 (5.0) | 1.27 (1.16, 1.38) | 1.40 (1.27, 1.56) | 2.15 (1.85) |
|  | Indicated | 26,671 (3.0) | 1,011 (5.2) | 1.62 (1.48, 1.78) | 1.74 (1.58, 1.93) | 2.87 (2.54) |

Doubly robust estimation: the *exposure* model included maternal age, birth year (continuous), SES, ethnicity/race, and MAR treatment, and the *outcome* model included maternal age, birth year (continuous), SES, parity, and ethnicity/race. The outcome model for preeclampsia and placenta previa also included gestational age. *SES: Socio economic status; MAR: Medically Assisted Reproduction; RR: Risk Ratio; CI: Confidence interval*

**Table S7. Covariate balance before and after matching using IPTW with standardized % of bias across covariates.**

|  | Unmatched (U) | Mean bias | |  | % Reduction | |
| --- | --- | --- | --- | --- | --- | --- |
| Variable | Matched (M) | **Endometriosis** | **No Endometriosis** | **% Bias** | **\|bias\|** |  |
| Smoking | U | 1.8349 | 1.8413 | -1.7 |  |  |
|  | M | 1.8349 | 1.8354 | -0.1 | 91.7 |  |
| MAR | U | 0.16831 | 0.02143 | 51.8 |  |  |
|  | M | 0.23034 | 0.23034 | 0.0 | 100 |  |
| Maternal age | U | 2.385 | 2.3552 | 2.8 |  |  |
|  | M | 2.7133 | 2.7151 | -0.2 | 94.2 |  |
| Gestational age | U | 2.9172 | 2.9457 | -9.1 |  |  |
|  | M | 2.9082 | 2.9125 | -1.4 | 84.9 |  |
| Birth year | U | 4.0269 | 4.3547 | -17.2 |  |  |
|  | M | 5.5537 | 5.5508 | 0.2 | 99.1 |  |
| Socio economic status | U | 2.9986 | 3.0023 | -0.3 |  |  |
|  | M | 3.2521 | 3.2515 | 0.0 | 82.7 |  |
| Parity | U | 1.0998 | 1.0428 | 4.7 |  |  |
|  | M | 1.1458 | 1.1479 | -0.2 | 96.2 |  |
| Ethnicity | U | 1.0894 | 1.1695 | -24 |  |  |
|  | M | 1.0986 | 1.1956 | -29.1 | -21.1 |  |

Mean Bias (U=14.0; M:3.9) Median Bias (U=6.9; M:0.2)
